# Fine-Tuning the Expression of Duplicate Genes by Translational Regulation in *Arabidopsis* and Maize

**DOI:** 10.3389/fpls.2019.00534

**Published:** 2019-05-08

**Authors:** Sishuo Wang, Youhua Chen

**Affiliations:** ^1^Chengdu Institute of Biology, Chinese Academy of Sciences, Chengdu, China; ^2^Department of Botany, Faculty of Science, The University of British Columbia, Vancouver, BC, Canada; ^3^School of Life Sciences, The Chinese University of Hong Kong, Sha Tin, Hong Kong

**Keywords:** gene duplication, genome evolution, plant genomics, translational regulation, expression evolution

## Abstract

Plant genomes are extensively shaped by various types of gene duplication. However, in this active area of investigation, the vast majority of studies focus on the sequence and transcription of duplicate genes, leaving open the question of how translational regulation impacts the expression and evolution of duplicate genes. We explored this issue by analyzing the ribo- and mRNA-seq data sets across six tissue types and stress conditions in *Arabidopsis thaliana* and maize (*Zea mays*). We dissected the relative contributions of transcriptional and translational regulation to the divergence in the abundance of ribosome footprint (RF) for different types of duplicate genes. We found that the divergence in RF abundance was largely programmed at the transcription level and that translational regulation plays more of a modulatory role. Intriguingly, translational regulation is characterized by its strong directionality, with the divergence in translational efficiency (TE) globally counteracting the divergence in mRNA abundance, indicating partial buffering of the transcriptional divergence between paralogs by translational regulation. Divergence in TE was associated with several sequence features. The faster-evolving copy in a duplicate pair was more likely to show lower RF abundance, which possibly results from relaxed purifying selection compared with its paralog. A considerable proportion of duplicates displayed differential TE across tissue types and stress conditions, most of which were enriched in photosynthesis, energy production, and translation-related processes. Additionally, we constructed a database TDPDG-DB (http://www.plantdupribo.tk), providing an online platform for data exploration. Overall, our study illustrates the roles of translational regulation in fine-tuning duplicate gene expression in plants.

## Introduction

One of the most prominent genomic differences between plants and other eukaryotes is the prevalence of duplicate genes in plant genomes (Panchy et al., 2016). Phylogenomic analyses have provided mounting evidence for recurrent episodes of ancient whole-genome duplication (WGD) throughout the evolutionary history of plants, with each WGD event superimposed on the genomic remnants of more ancient ones (Bowers et al., 2003; Adams and Wendel, 2005; Van De Peer et al., 2009; Jiao et al., 2011; Ruprecht et al., 2017; Ren et al., 2018; Reuscher et al., 2018). In addition, duplicate genes can be generated from other mechanisms (e.g., tandem duplication), which, together with WGD, contribute to the great preponderance of duplicate genes in plant genomes (Panchy et al., 2016; Wang et al., 2018). Gene duplication provides an enormous reservoir of new genes for the innovation of functions and phenotypic traits, and is a primary force in driving genome evolution of flowering plants (Flagel and Wendel, 2009; Schranz et al., 2012; Dai et al., 2014; Soltis and Soltis, 2016; Jiao, 2018).

Investigating gene duplication and the evolutionary fates of genes after duplication is of fundamental importance in the understanding of plant genomes. One of the most important aspects of this issue is to understand expression conservation and divergence between paralogs. Several models have been proposed to interpret this issue (Zhang, 2003). Paralogs may subdivide their ancestral functions such that both copies become essential and are selectively retained (subfunctionalization) (Hughes, 1994; Force et al., 1999). Alternatively, one duplicate copy might evolve novel expression patterns or functions (neofunctionalization) (Ohno, 1970; Hughes et al., 2014). Counterbalancing these ideas is the gene dosage hypothesis, where both paralogs are subjected to constraints on dosage balance and show high conservation in sequence and expression (Edger and Pires, 2009; Conant et al., 2014). These evolutionary scenarios are not mutually exclusive [e.g., constraints on gene dosage may provide long enough time for duplicates to diverge in function (Force et al., 1999; Conant et al., 2014; Vaattovaara et al., 2019)], but their relative contributions remain to be explored in depth.

To date, our understanding of the expression between duplicate genes has been largely guided by studies using transcriptomic data. By microarray and RNA-seq analysis, many studies have demonstrated the divergence between paralogs in transcription (Li et al., 2005; Ganko et al., 2007; Roulin et al., 2013). However, gene expression is a dynamic process including transcription, translation, and protein turnover; transcript abundance may not always be biologically meaningful (Vogel and Marcotte, 2012; Bailey-Serres, 2013; Shah et al., 2013; Gamm et al., 2014; Liu et al., 2016). For example, several studies have estimated the divergence in microRNA regulation between paralogs in various plant species, and suggested its contributions to expression divergence between duplicates (Guo et al., 2008; Sun et al., 2015; Wang and Adams, 2015). It was also shown that more than 85% of paralogs in *Arabidopsis* show divergence in nonsense-mediated decay induced by alternative splicing (Tack et al., 2014). These studies reveal the crucial roles of post-transcriptional regulation between paralogous genes.

Translation is one of the most energy-consuming processes in cell (Buttgereit and Brand, 1995; Lynch and Marinov, 2015). As such, fine regulation of translation is very important in regulating the level of gene expression and protein synthesis to the actual needs. Translational regulation might particularly be important for plants considering their complex translational apparatus and additional genetic systems in chloroplasts and mitochondria (Ferrando et al., 2017). However, for a long period, our understanding of translational regulation is limited to a small number of genes, and the link between transcript abundance and protein synthesis still waits to be understood (Koh et al., 2012; Hu et al., 2013). Recently, ribosome profiling technology (ribo-seq) (Ingolia et al., 2009) has emerged as a powerful method to identify translating mRNAs, which provides an alternative and robust way to assess gene expression and allows for identification of translational regulation on a genome-wide scale (Ingolia, 2014; Merchante et al., 2017). Using ribo-seq and its related technologies, several studies have revealed the important roles of translational regulation of gene expression in plants, suggesting that the functional patterns of the expression of many genes may not be established until translation (Juntawong et al., 2014; Lei et al., 2015; Hsu et al., 2016; Bai et al., 2017). Coate et al. (2014) analyzed the translatome of a recently formed polyploid *Glycine*. This study revealed rapid changes in translation shortly after polyploidization, suggesting a previously unappreciated role of translational regulation in reducing expression differences between polyploid and parents. Although the results of this study are very encouraging, it is worth noting that the vast majority of modern plants are diploidized paleopolyploids (Panchy et al., 2016; Pont and Salse, 2017). Mechanisms of translational regulation in recently formed polyploids might not apply to other modern plants where on average 65% of genes in the genome are duplicates that have been retained for millions of years (Panchy et al., 2016). Considering the importance of translation in gene expression and the prevalence of duplicates in plant genomes, a thorough study of translational regulation between paralogs in plants is urgently needed.

To investigate translational divergence between paralogs and its impacts on duplicate gene evolution, we carried out a comprehensive analysis of translational regulation of paralogs derived from WGD as well as tandem duplication in *Arabidopsis thaliana* and maize (*Zea mays*) by integrating six paired ribo-/mRNA-seq data sets from different tissue types and stress conditions. We analyzed the divergence between the RF abundance (i.e., the abundance of ribosome-associated reads mapped in ribo-seq) and mRNA abundance for duplicate genes. We found that while the divergence in RF abundance is mainly underlined at the transcriptional level, translational regulation tends to buffer transcriptional divergence between paralogs. In addition, we explored tissue- and stress-specific translational regulation between paralogs. We also analyzed the relationship between translational regulation and evolutionary rate for duplicates. Finally, we present on online database TDPDG-DB^1^ for data exploration.

## Materials and Methods

### Selection of Duplicate Gene Pairs

We obtained the 3,183 pairs of *Arabidopsis* duplicates derived from the alpha WGD identified in Bowers et al. (2003). Maize WGD duplicates and their information regarding subgenome were retrieved from Schnable et al. (2011). Tandem duplicates were selected using the following procedure (Zou et al., 2009; Liu et al., 2011). First, we clustered duplicate genes into tandem clusters if they (i) belong to the same family, (ii) are separated by at most 10 genes, and (iii) are located within 100 kb on the same chromosome. Then, for tandem gene clusters with more than two members, two genes were randomly chosen as the representative duplicate genes of the cluster. In total, a set of 2002 and 1706 pairs of tandem duplicates were collected for *Arabidopsis* and maize, respectively. The rest genes were classified as other types of duplicates, if they had non-self BLASTP hits with E-value lower than or equal to 1e-10, or singletons, if they had no non-self BLASTP hits with *E*-values less than or equal to 1e-3 (Wang and Adams, 2015). For genes with multiple isoforms, only the one with the longest sequence was selected as the representative. Sequence format conversion and processing were conducted with BEDOPS v2.4.14 (Neph et al., 2012), and custom scripts written in Ruby (Goto et al., 2010).

### mRNA-Seq and Ribo-Seq Data Sets and Read Mapping

We collected deep-sequencing mRNA- and ribo-seq data sets of root and shoot from Hsu et al. (2016) and seedlings under normal and sublethal hypoxia stress conditions from Juntawong et al. (2014) for *A. thaliana* (Supplementary Table S1). We retrieved mRNA- and ribo-seq data of maize seedling under normal and drought conditions from Lei et al. (2015). mRNA- and ribo-seq data sets of the same tissue type or stress condition were generated from the same study. All of these data sets have at least two biological replicates.

Reads shorter than 20 nucleotides were removed before mapped to the genome. Terminal nucleotides with the sequencing quality less than or equal to 20 were trimmed by Cutadapt v1.3 (Martin, 2011). Reads were mapped to the reference genome with STAR v2.4.2a (Dobin et al., 2013) with the parameters “STAR –genomeDir index –readFilesIn fastqs –outSAMtype BAM SortedByCoordinate –alignIntronMax 25000 –outSAMstrandField intronMotif”.

### Calculation of the Relative Divergence of RF and mRNA Abundance

Fragments per kilobase per million mapped fragments (FPKM) for each gene was calculated with Cufflinks v2.2.1 (Trapnell et al., 2014). Since all of the ribo- and mRNA-seq data sets used in this study have multiple replicates, the expression level of each gene was averaged over all replicates in subsequent analysis. Because very lowly expressed genes are likely to be artifacts (Bhargava et al., 2014), we filtered out genes with average FPKM lower than 0.1 (Chettoor et al., 2014; Joag et al., 2016; Sun et al., 2018).

The signed relative divergence of RF or mRNA abundance is calculated as (X2 - X1)/(X1 + X2), where X1 and X2 represent the FPKM value in the ribo- or mRNA-seq of the first and the second gene in a pair of duplicates, respectively, as calculated for the relative divergence of sequence evolutionary rate shown above. This measure quantifies the relative RF or mRNA abundance difference between a pair of paralogs by normalizing the overall RF or mRNA abundance of the pair (Conant and Wagner, 2003; Kim and Yi, 2006; Keller and Yi, 2014).

### Calculation of Translational Efficiency

TE was calculated as ribo-seq FPKM/mRNA-seq FPKM as previously described (Ingolia et al., 2009), and has been used as a proxy of the translational speed and accuracy in many studies (Gerashchenko et al., 2012; Juntawong et al., 2014; Lei et al., 2015; Wang et al., 2015). The relative divergence of TE was calculated as (X2 - X1)/(X1 + X2), where X1 and X2 represent the TE of the first and the second paralog, respectively.

Differentially translated genes across tissue types or stress conditions were identified using RiboDiff, which utilizes generalized linear model strategies to detect genes showing differential TE between data sets (Zhong et al., 2017). To perform analysis with RiboDiff, uniquely mapped reads from each mRNA-seq and ribo-seq dataset were counted for each gene using featureCounts implemented in subread v1.4.6 (Liao et al., 2013). Genes with FDR-adjusted (Benjamini and Hochberg’s method) *P*-value lower than 0.05 were flagged as genes that display differential TE (Zhong et al., 2017).

### Calculation of Sequence Divergence and Identification of Asymmetric Evolution Between Duplicate Genes

Calculation of the relative amino acid divergence and identification of asymmetric evolution between duplicate genes followed the procedure described in previous studies (Blanc and Wolfe, 2004; Liu et al., 2011). Protein sequences for each duplicate gene pair were aligned using MUSCLE v3.8.31 (Edgar, 2004), which was then used as an guide to generate codon alignment using PAL2NAL (Suyama et al., 2006). To identify orthologs, we collected protein sequences of *Carica papaya* and *Vitis vinifera*, as used in Liu et al. (2011), and added protein sequences from *Theobroma cacao, Citrus sinensis, Fragaria vesca, Ricinus communis* as the outgroup. For maize, *Setaria italica, Sorghum bicolor, Oryza sativa*, and *Oropetium thomaeum* were selected as outgroup species. These species were chosen as the outgroup since they split from the lineages to *Arabidopsis* or maize before the WGD event analyzed in this study, and do not show any evidence for WGD after their split (Ren et al., 2018).

We employed codeml implemented in PAML v4.7 (Yang, 2007) to determine the non-synonymous (*K*_a_) and synonymous substitution (*K*_s_) rate for all duplicate gene pairs. Triplets where the value of *K*_a_ between the paralogs in *Arabidopsis* (or maize) was larger than that between the paralogs in *Arabidopsis* (or maize) and the ortholog in the outgroup species were discarded (Blanc and Wolfe, 2004). The relative divergence of *K*_a_ (or *K*_a_/*K*_s_) was defined as (X2 - X1)/(X1 + X2), where X1 and X2 stand for the *K*_a_ (or *K*_a_/*K*_s_) for the two paralogs, respectively (Conant and Wagner, 2003; Fares et al., 2013). Then we computed the log likelihood (lnL) of the triplets under two competing evolutionary models (Blanc and Wolfe, 2004; Liu et al., 2011). The first model assumes that evolutionary rates are unconstrained (i.e., asymmetric evolution), and the second model assumes that duplicate genes evolve at clock-like rates (i.e., symmetric evolution). To test whether the model of asymmetric evolution fits better than the model of symmetric evolution, we applied the likelihood ratio test (LRT). In brief, twice the difference of the log likelihood under the two models [2ΔlnL, where ΔlnL = lnL(no constraint) - lnL(clock)] was compared against a chi-square distribution with one degree of freedom. Duplicate gene pairs with FDR-adjusted (Benjamini and Hochberg’s method) *P*-values lower than 0.05 were determined to show asymmetrical protein sequence evolution.

### Gene Ontology Analysis

Gene Ontology enrichment was analyzed using topGO implemented in the PlantRegMap platform (Jin et al., 2017). Those with FDR-adjusted *P*-value lower than 0.05 in the Fisher’s exact test were considered as overrepresented GO categories.

### RNA Structure Prediction

We used RNAfold, a core program from Vienna RNA v1.8.5 (Gruber et al., 2008) to predict the minimum free energy RNA secondary structure at default temperature 37°C with default parameters.

## Results

### Translational Buffering of Transcriptional Divergence Between Duplicates

**FIGURE 1:**
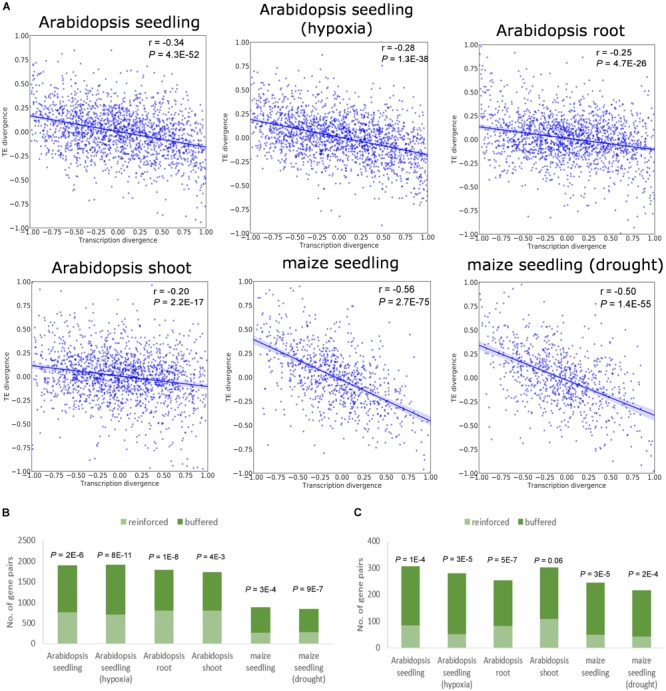
Relationship between mRNA abundance divergence and TE divergence for WGD duplicates. **(A)** Scatter plots showing the correlation between the relative divergence in mRNA abundance and the relative divergence in TE. The Pearson’s correlation coefficient and *P*-value are indicated. The shaded areas represent 95% confidence interval in both plots. **(B,C)** Numbers of gene pairs whose transcriptional divergence decreases (referred to as “buffered”) or increases (referred to as “reinforced”) at the translational level for all WGD duplicates **(B)** and those with TE fold difference ≥ 2 **(C)**. *P*-values derived from the binomial test are shown above the bar.

Translational regulation could act in two ways for duplicate genes, either in the same direction as transcriptional regulation to amplify the mRNA abundance divergence, or in the opposite direction to buffer the expression divergence established at the transcriptional level. To determine the directionality of translational regulation for duplicate genes, we calculated the signed relative divergence of mRNA abundance and TE as (X2 - X1)/(X1 + X2), where X1 and X2 denote the mRNA abundance or TE for the two paralogs, respectively (see also section “Materials and Methods”). Defined as the amount of RF normalized to underlying mRNA abundance (Ingolia et al., 2009), TE is widely used as an indicator of the propensity of mRNA to undergo translation (see also section “Materials and Methods”). The relationship between the divergence of mRNA abundance and TE for WGD duplicates is depicted in Figure 1A. As shown in the figure, mRNA divergence displayed a strong correlation with TE divergence between WGD duplicates in both species regardless of tissue types and stress conditions (Figure 1A). Similarly, tandem duplicates exhibited significant negative correlation between mRNA abundance divergence and TE divergence in all analyzed data sets (Supplementary Figure S1A). The above results indicate that the duplicate copy with the higher mRNA abundance is more likely to display lower TE compared with its paralog.

To further illustrate this pattern, we calculated the proportions of duplicate genes where mRNA abundance divergence was reduced and increased at the translational level, respectively. The divergence in mRNA abundance for an average of 58 and 68% of WGD duplicates in *Arabidopsis* and maize, respectively, was reduced when measured at the level of RF abundance, significantly higher than those with increased expression divergence when measured by RF abundance (Figure 1B). Moreover, we restricted the analysis to duplicates that displayed at least twofold difference in TE. With 72 and 80% of WGD duplicates exhibiting reduced expression divergence in RF abundance in *Arabidopsis* and maize, respectively, the pattern was even stronger (Figure 1C). The similar pattern was found for tandem duplicates (Supplementary Figures S1B,C).

We further investigated sequence features that are potentially associated with TE divergence between paralogs. For both WGD and tandem duplicates in maize, the GC content of CDS was positively correlated with the relative divergence of TE, and the length and minimal free energy of the predicted secondary structure of 3′ UTR displayed negative correlation with TE divergence (Supplementary Tables S2, S3). In contrast, for *Arabidopsis* duplicates, the divergence of examined sequence features exhibited weak correlations with TE divergence (Supplementary Tables S2, S3). This suggests that multiple factors may act together on translational regulation and that the sequence features affecting TE divergence vary across species (Lei et al., 2015; Bai et al., 2017; Zhao et al., 2017).

### Expression Divergence Between Duplicates Is Mainly Underlined by Transcript Abundance

**FIGURE 2:**
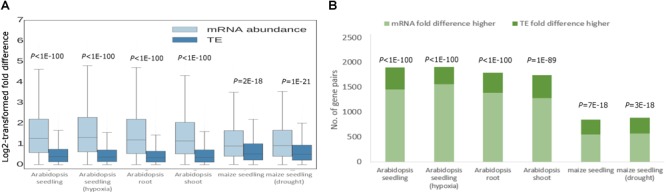
Relative contributions of transcriptional and translational regulation to RF abundance divergence of WGD duplicates. **(A)** Log2-transformed fold differences in mRNA abundance and TE between WGD paralogs. Boxes extend from the first quartile (Q1) to the third quartile (Q3). The median is shown by a line inside the box. Whiskers extend to ± 1.5 interquartile range (IQR). *P*-values derived from the Wilcoxon signed-rank test are shown. **(B)** Numbers of WGD duplicate pairs with higher fold difference in mRNA abundance than TE and with higher fold difference in TE than mRNA abundance. *P*-values derived from the binomial test are indicated.

Next, we dissected the relative contributions of transcriptional regulation and translational regulation to the divergence in RF abundance by comparing the fold differences of mRNA abundance and TE for each pair of paralogs (Gerashchenko et al., 2012). In all analyzed tissue types and stress conditions, the fold difference of mRNA abundance between paralogs was significantly greater than that of TE for both WGD duplicates (Figure 2A) and tandem duplicates (Supplementary Figure S2A). Specifically, in *Arabidopsis*, an average of 75% of WGD duplicates exhibited higher fold difference of mRNA abundance than that of TE (Figure 1B). The proportion stood at 65%, on average, for maize WGD duplicates (Figure 1B). Similar patterns were found for tandem duplicates (Supplementary Figure S2). In addition, we did not detect significant difference in the proportion of duplicates with greater TE fold difference between WGD and tandem duplicates (Figure 2 and Supplementary Figure S2). The results indicate that the divergence in RF abundance was in general underlined by transcript abundance divergence for most duplicates in *Arabidopsis* and maize.

### The Faster-Evolving Paralog Copy Is More Likely to Exhibit a Lower RF Abundance

**FIGURE 3:**
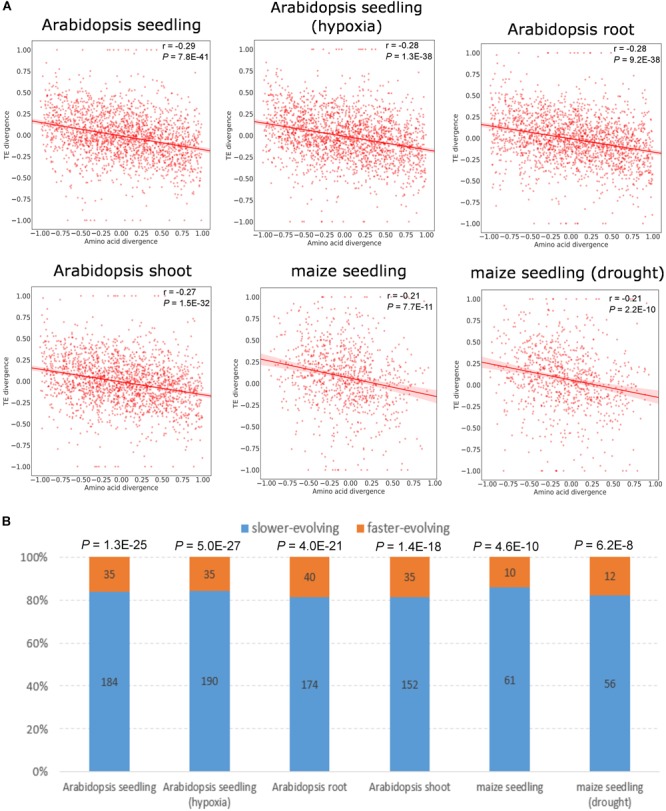
Relationship between RF abundance divergence and amino acid divergence for WGD duplicates. Amino acid divergence is measured as non-synonymous substitution rate (*K*_a_), as widely used in prior studies (Li et al., 2005; Ganko et al., 2007; Hakes et al., 2007). **(A)** Correlation between the RF abundance divergence and amino acid sequence divergence. The Pearson’s correlation coefficient and *P*-value are indicated. The shaded areas represent 95% confidence interval in both plots. **(B)** Proportion of the paralog copy with the higher RF abundance between the slower- and faster-evolving copy in a duplicate pair. The *y*-axis represents the proportion of the paralog with the higher RF abundance. Bars in blue and yellow denote the slower-evolving and faster-evolving paralog copy, respectively. Numbers of duplicate pairs are given on the bar. *P*-values derived from a binomial test are shown above the bar.

To explore the association between expression divergence and sequence divergence for duplicate genes, we calculated the relative divergence in RF abundance and amino acid sequence between paralogs (see section “Materials and Methods” and Supplementary Table S4). We observed significant negative correlation of the relative divergence between RF abundance and amino acid sequence in all data sets except for maize tandem duplicates (Figure 3A and Supplementary Figure S3A). Further, we restricted the analysis to duplicates showing at least twofold difference in RF abundance and asymmetric sequence evolution (see section “Materials and Methods”), and the same pattern held true (Figure 3B and Supplementary Figure S3B). Similar to RF abundance divergence, mRNA abundance divergence was negatively correlated with protein sequence divergence (Supplementary Tables S5, S6). However, we detected little correlation between TE divergence and amino acid divergence for duplicate genes (Supplementary Tables S5, S6). Thus, the faster-evolving copy was more likely to exhibit lower RF abundance than its slower-evolving paralog, which is mainly determined at the transcription level.

We further calculated *K*_a_/*K*_s_ (the ratio of non-synonymous substitution rate and synonymous substitution rate) to examine the role of selection in the expression divergence between paralogs, as commonly used in prior studies (Jordan et al., 2004; Kondrashov, 2012; Hamaji et al., 2018). The *K*_a_/*K*_s_ divergence was negatively correlated with RF abundance divergence, although the correlation was in general weaker than that between amino acid divergence and RF abundance divergence (Supplementary Tables S5, S6). The *K*_a_/*K*_s_ of the vast majority of the paralog copy with the higher RF abundance ranged from 0.1–0.4, and only a few displayed *K*_a_/*K*_s_ higher than 1.0, which might be suggestive of positive selection (Supplementary Figure S4). This implies that the more lowly translated paralog tended to be under less selective constraints compared with its paralog.

### Tissue- and Stress-Specific Translational Regulation of Duplicates

**FIGURE 4:**
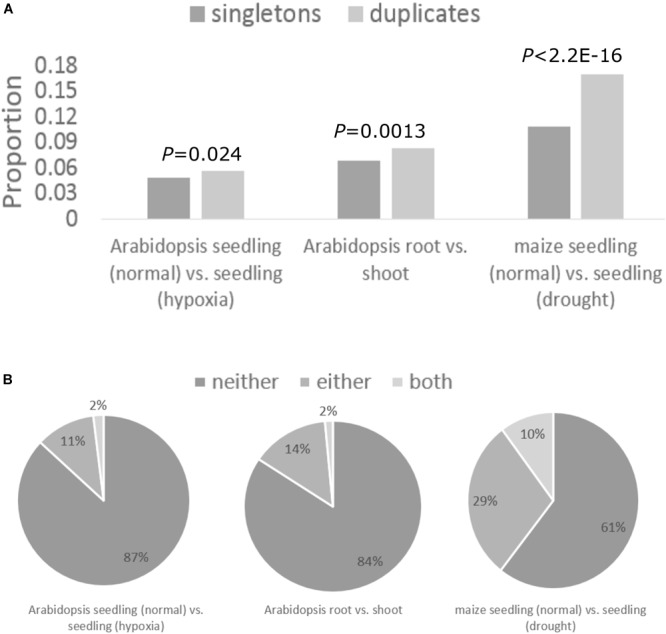
Tissue- and stress-specific translational regulation of WGD duplicates. **(A)** Proportions of duplicates and singletons that showed differential TE between tissue types or under stress conditions. *P*-values obtained from the chi-squared test are shown above the bar. **(B)** Pie charts showing the proportion of WGD duplicates where neither copy (referred to as “neither”), one copy (referred to as “either”), and both copies (referred to as “both”) showed differential TE.

We then asked how translational regulation of duplicates varies across tissue types and in response to abiotic stress. We identified genes with differential TE across tissue types or stress conditions using RiboDiff between the normal and hypoxia condition for *Arabidopsis* seedling, between *Arabidopsis* root and shoot, and between the normal and drought condition for maize seedling (Supplementary Table S7; see section “Materials and Methods”). Genes with differential TE across tissue types or stress conditions were enriched in duplicate genes (i.e., WGD duplicates, tandem duplicates, and other types of duplicates; see section “Materials and Methods”) compared with singletons (Figure 4A). This suggests that translational regulation preferentially regulates the expression of duplicates over singletons in plants. On average, 13 and 29% of WGD duplicates had one copy differentially translated in *Arabidopsis* and maize, respectively (Figure 4B). For 2 and 10% of WGD duplicates in *Arabidopsis* and maize, respectively, both copies showed differential TE between tissue types or stress conditions (Figure 4B). The higher proportion of duplicates with differentially translated genes in maize hints more translational regulation in maize than *Arabidopsis*, consistent with above results.

Gene ontology analysis revealed that the majority of duplicate genes with differential TE across tissues or stresses are those targeted to ribosome or chloroplast, and are involved in peptide biosynthesis, rRNA processing, photosynthesis, energy production, and protein degradation (Supplementary Table S8). As translation is one of the most energy-consuming processes in the cell (Buttgereit and Brand, 1995; Lynch and Marinov, 2015), the translational regulation of genes in these GO categories may serve as an energy conservation mechanism and help plants rapidly respond to environmental changes (Juntawong et al., 2014; Lei et al., 2015; Toribio et al., 2016; Wu et al., 2018). Of particular interest are genes encoding ribosomal proteins or proteins participating in ribosome biogenesis, because these genes are directly related to the translation of the entire transcriptome. This finding sparks the idea that, in response to stresses, plants may operate gene expression network by regulating the translation of translational apparatus, which in turn facilitates the translational regulation of the cell through a positive feedback (Juntawong et al., 2014). The partition of expression at the translational level adds a new layer of regulation for duplicate genes, which might facilitate functional divergence and long-term retention of both paralogs.

### TDPDG-DB: An Online Database for Translational Divergence of Plant Duplicate Genes

To compile an archive of the translational regulation of plant duplicate genes and facilitate their research, we developed an online database TDPDG-DB (Translational Divergence of Plant Duplicate Genes Database^2^), which comprises all analyzed data sets in this study and makes them easily accessible to researchers (Supplementary Figure S5A). Through the “Search” interface, researchers can easily search for genes of interest by the data set, type of gene duplication, gene locus name, the fold difference of RF abundance and TE level, or any combination of the above (Supplementary Figure S5B). Users can further view the details of the translational divergence as well as other related information of each paralog pair by clicking on the gene name in the search results (Supplementary Figure S5C). Additionally, users can download the original data deposited in the database by clicking on “Download” in the main toolbar.

## Discussion

Gene expression is a complex stepwise process involving regulation at many layers. Although expression evolution between duplicate genes has been well studied at the transcriptional level, much less attention has been paid to translation, one of the most energy-consuming processes in the cell (Buttgereit and Brand, 1995; Lynch and Marinov, 2015). The negative correlation between the divergence of mRNA abundance and TE shown in our study reveals that the paralog copy with higher mRNA abundance tended to display lower TE. Therefore, translational regulation of duplicate genes more often counteracts than follows the divergence in mRNA abundance, which partially compensates for their divergence in mRNA abundance. Our current understanding of expression relies heavily on transcriptomic data (reviewed in Panchy et al., 2016). Coate et al. (2014) analyzed the translatome of a recently formed polyploid *Glycine* (∼0.1 Mya), showing that changes in translation changed shortly after polyploidization to reduce expression differences between the polyploid and parents. This, together with our study, reveals the widespread impact of translational buffering on duplicate gene expression in *Arabidopsis* and maize, suggesting that the extent to which the expression and function of duplicate genes diverge is likely overestimated when measured only at the transcription level. As expression similarity is an important indicator of functional similarity (Blanc and Wolfe, 2004; Wagner, 2005), post-transcriptional buffering of expression divergence might reduce the functional divergence between paralogs. Though divergence in expression and function is often thought to be crucial to the retention of duplicate genes, functional redundancy to some extent between paralogs may also have its benefits. Studies have shown that redundancy between duplicate genes could lead to buffering effect against null mutations, thereby increasing the robustness of the gene regulatory network (Gu et al., 2003; Dean et al., 2008; Diss et al., 2014; Keane et al., 2014). In addition, for some duplicate genes, especially those in the same complex or involved in the same pathways, it may be important to maintain the right dosage balance (Edger and Pires, 2009; Birchler and Veitia, 2012; Wang and Chen, 2018; Gout et al., 2019). In this regard, by buffering the expression divergence established at the transcriptional level, translational regulation might fine-tune the expression level of both paralog copies, and help them better maintain the appropriate gene dosage.

The idea of translational buffering can be illustrated by a pair of WGD-derived duplicates involved in signaling cascades, MAPKKK17 (AT2G32510) and MAPKKK18 (AT1G05100). While the two paralogs were shown to function redundantly in root, they displayed about fourfold transcriptional divergence (Danquah et al., 2015; Li et al., 2017). The present study suggests that the divergence was compensated by their different translational efficiencies^3^, which results in nearly no difference in RF abundance and might contribute to functional redundancy of the duplicates.

Note, however, that despite its global trend to buffer divergence in mRNA abundance, the impact of translational regulation for plant duplicate genes should not be over-exaggerated. As clearly shown in the present study, the divergence in TE is of markedly smaller scale than the divergence of mRNA abundance between paralogs (Figure 2 and Supplementary Figure S2). The medians of the fold difference of mRNA abundance were roughly twice and 1.5 times the fold difference of RF abundance in *Arabidopsis* and maize, respectively (Figure 2 and Supplementary Figure S2). Additionally, despite the general pattern of translational buffering, for many paralogous genes, translational regulation likely led to more divergent expression between them (Figure 1 and Supplementary Figure S1). Hence, we argue that the expression divergence between paralogs is mainly set up at the transcription level, whereas translational regulation plays more of a modulatory role to fine-tune the expression of plant duplicate genes.

In addition, we showed that the paralog with the higher RF abundance in a duplicate pair tended to evolve more slowly and be under more selective constraints than the other copy. This is generally consistent with previous studies for duplicate genes (Pal et al., 2001; Kim and Yi, 2006) [see also Duret and Mouchiroud (2000); Nuzhdin et al. (2004), Subramanian and Kumar (2004); Zhang and He (2005), Lemos et al. (2005), and Drummond et al. (2006) for genes that are not limited to duplicates]. The fast evolution might lead to changes in regulatory elements, which in turn triggers the decrease in expression (Arsovski et al., 2015). Alternatively, the reduction of expression could occur first, which relaxes the selection pressure against amino acid change and allows for the accelerated sequence evolution of the duplicate copy (Zhang, 2003). If selection on precise gene expression mainly acts on protein abundance, translational buffering might lead to more tolerance to the variation in transcription, as phenotypic effects in mRNA abundance variation between paralogs could be masked at the translational level (Castelo-Szekely et al., 2017). It is possible that the networks of transcriptional and translational regulation diverge by genetic compensation, such that mutations in translation might counteract the effects of mutations in transcription or vice versa, resulting in translational buffering of expression divergence between paralogs (Mcmanus et al., 2014). This could be achieved by differences in sequence features of UTR and CDS, as shown in this and previous studies (Lei et al., 2015; Hsu et al., 2016; Zhao et al., 2017).

Translational regulation of genes is starting to be appreciated, but is still poorly understood. To facilitate the research of translational regulation for plant duplicate genes, we developed an online database TDPDG-DB^4^. We hope that this database can serve as a useful platform for researchers in related fields. Future studies of molecular mechanisms of changes in TE will provide more insights into the translational divergence of duplicate genes. It would also be important to examine whether patterns found in this study hold true in other species, and apply comparative genomics to assess the evolutionary conservation of translational regulation across lineages.

## Author Contributions

SW and YC conceived the study. SW performed the analysis. SW and YC analyzed the data and wrote the manuscript.

## Conflict of Interest Statement

The authors declare that the research was conducted in the absence of any commercial or financial relationships that could be construed as a potential conflict of interest.

## Footnotes

1 http://plantdupribo.tk

2 http://plantdupribo.tk

3 http://plantdupribo.tk/single_gene.php?pair=AT1G05100-AT2G32510&tissue=root_Hsu_2016

4 http://plantdupribo.tk

## Supplementary Material

FIGURE S1 Relationship between mRNA abundance divergence and TE divergence for tandem duplicates. **(A)** Scatter plots showing the correlation between the relative divergence in mRNA abundance and the relative divergence in TE. The Pearson’s correlation coefficient and *P*-value are indicated. The shaded areas represent 95% confidence interval in both plots. **(B,C)** Numbers of gene pairs whose transcriptional divergence decreases (referred to as “buffered”) or increases (referred to as “reinforced”) at the translational level for all tandem duplicates **(B)** and those with TE fold difference ≥ 2 **(C)**. *P*-values derived from the binomial test are shown above the bar.

FIGURE S2 Relative contributions of transcriptional and translational regulation to RF abundance divergence of tandem duplicates. **(A)** Log2-transformed fold differences in mRNA abundance and TE between paralogs. Boxes extend from the first quartile (Q1) to the third quartile (Q3). The median is shown by a line inside the box. Whiskers extend to ±1.5 interquartile range (IQR). *P*-values derived from the Wilcoxon signed-rank test are shown. **(B)** Numbers of tandem duplicate pairs with higher fold difference in mRNA abundance than TE and with higher fold difference in TE than mRNA abundance. *P*-values derived from the binomial test are indicated.

FIGURE S3 Relationship between RF abundance divergence and amino acid divergence for tandem duplicates. **(A)** Correlation between the RF abundance divergence and amino acid sequence divergence. The Pearson’s correlation coefficient and *P*-value are indicated. The shaded areas represent 95% confidence interval in both plots. **(B)** Proportion of the paralog copy with the higher RF abundance between the slower- and faster-evolving copy in a duplicate pair. The *y*-axis represents the proportion of the paralog with the higher RF abundance. Bars in blue and yellow denote the slower-evolving and faster-evolving paralog copy, respectively. Numbers of duplicate pairs are given on the bar. *P*-values derived from a binomial test are shown above the bar.

FIGURE S4 Distribution of the value of *K*_a_/*K*_s_ of the paralog copy with the higher RF abundance for WGD **(A)** and tandem duplicates **(B)**.

FIGURE S5 The database for translational divergence of plant duplicate genes. **(A)** Homepage of the database. **(B)** The ‘Search’ interface of the database. **(C)** An examples of the search results.

TABLE S1 Mapping information of all analyzed ribo-seq/mRNA-seq data sets.

TABLE S2 Correlation between the relative divergence of TE and the relative divergence of sequence features in CDS and UTR for WGD duplicates.

TABLE S3 Correlation between the relative divergence of TE and the relative divergence of sequence features in CDS and UTR for tandem duplicates.

TABLE S4 Sources of genomics information of plants used in the study.

TABLE S5 Correlation of the relative divergence between mRNA abundance, TE, and *K*_s_, and between RF abundance and *K*_a_/*K*_s_ for WGD duplicates. The Pearson’s correlation coefficient and *P*-value are shown.

TABLE S6 Correlation of the relative divergence between mRNA abundance, TE, and *K*_s_, and between RF abundance and *K*_a_/*K*_s_ for tandem duplicates. The Pearson’s correlation coefficient and *P*-value are shown.

TABLE S7 Identification of genes with differential TE across tissue types or stress conditions using RiboDiff.

TABLE S8 Gene ontology (GO) categories enriched in duplicate genes with differential TE across tissue types or stress conditions.

## Abbreviations

GO: Gene Ontology
*K*_a_: non-synonymous substitution rate
*K*_s_: synonymous substitution rate
RF: ribosome footprint
TE: translational efficiency
WGD: whole-genome duplication
