# Supplementary material for: Fine-Tuning the Expression of Duplicate Genes by Translational Regulation in Arabidopsis and Maize
Source: Front Plant Sci. 2019 May 8;10:534. doi: 10.3389/fpls.2019.00534 (PMC6530396; doi:10.3389/fpls.2019.00534)
Supplement: FIGURE S1 — Relationship between mRNA abundance divergence and TE divergence for tandem duplicates. (A) Scatter plots showing the correlation between the relative divergence in mRNA abundance and the relative divergence in TE. The Pearson’s correlation coefficient and P-value are indicated. The shaded areas represent 95% confidence interval in both plots. (B,C) Numbers of gene pairs whose transcriptional divergence decreases (referred to as “buffered”) or increases (referred to as “reinforced”) at the translational level for all tandem duplicates (B) and those with TE fold difference ≥ 2 (C). P-values derived from the binomial test are shown above the bar. [file Data_Sheet_1.docx]

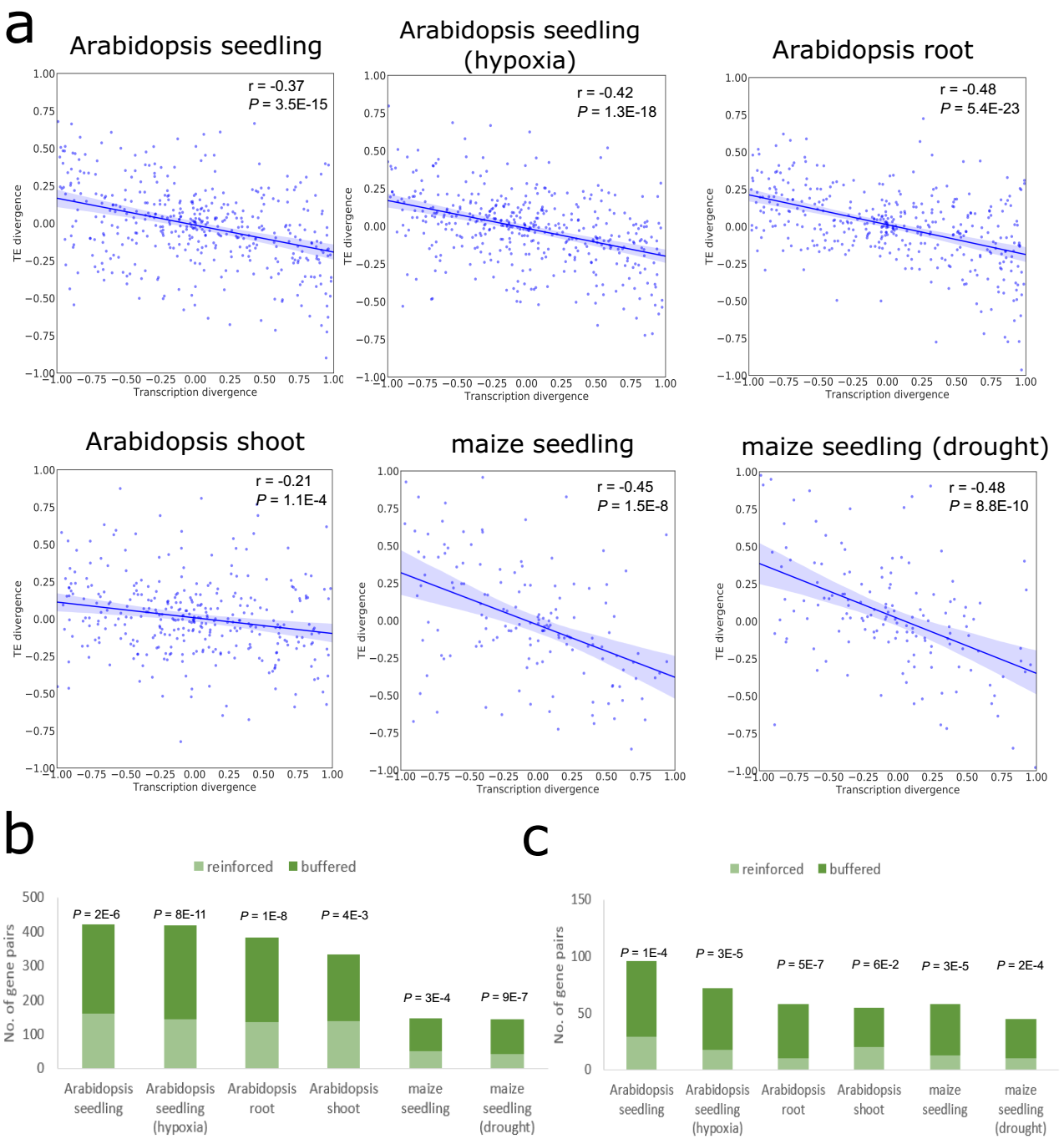


**Fig. S1.** Relationship between mRNA abundance divergence and TE divergence for tandem duplicates. (a) Scatter plots showing the correlation between the relative divergence in mRNA abundance and the relative divergence in TE. The Pearson’s correlation coefficient and *P*-value are indicated. The shaded areas represent 95% confidence interval in both plots. (b, c) Numbers of gene pairs whose transcriptional divergence decreases (referred to as “buffered”) or increases (referred to as “reinforced”) at the translational level for all tandem duplicates (b) and those with TE fold difference ≥ 2 (c). *P*-values derived from the binomial test are shown above the bar.


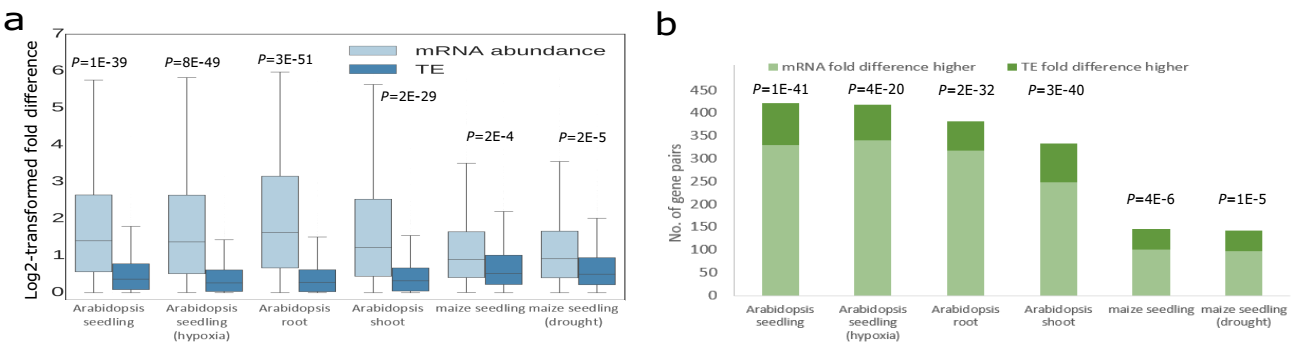


**Fig. S2.** Relative contributions of transcriptional and translational regulation to RF abundance divergence of tandem duplicates. (a) Fold differences in mRNA abundance and TE between paralogs. Boxes extend from the first quartile (Q1) to the third quartile (Q3). The median is shown by a line inside the box. Whiskers extend to ± 1.5 interquartile range (IQR). *P*-values derived from the Wilcoxon signed-rank test are shown. (b) Numbers of tandem duplicate pairs with higher fold difference in mRNA abundance than TE and with higher fold difference in TE than mRNA abundance. *P*-values derived from the binomial test are indicated.


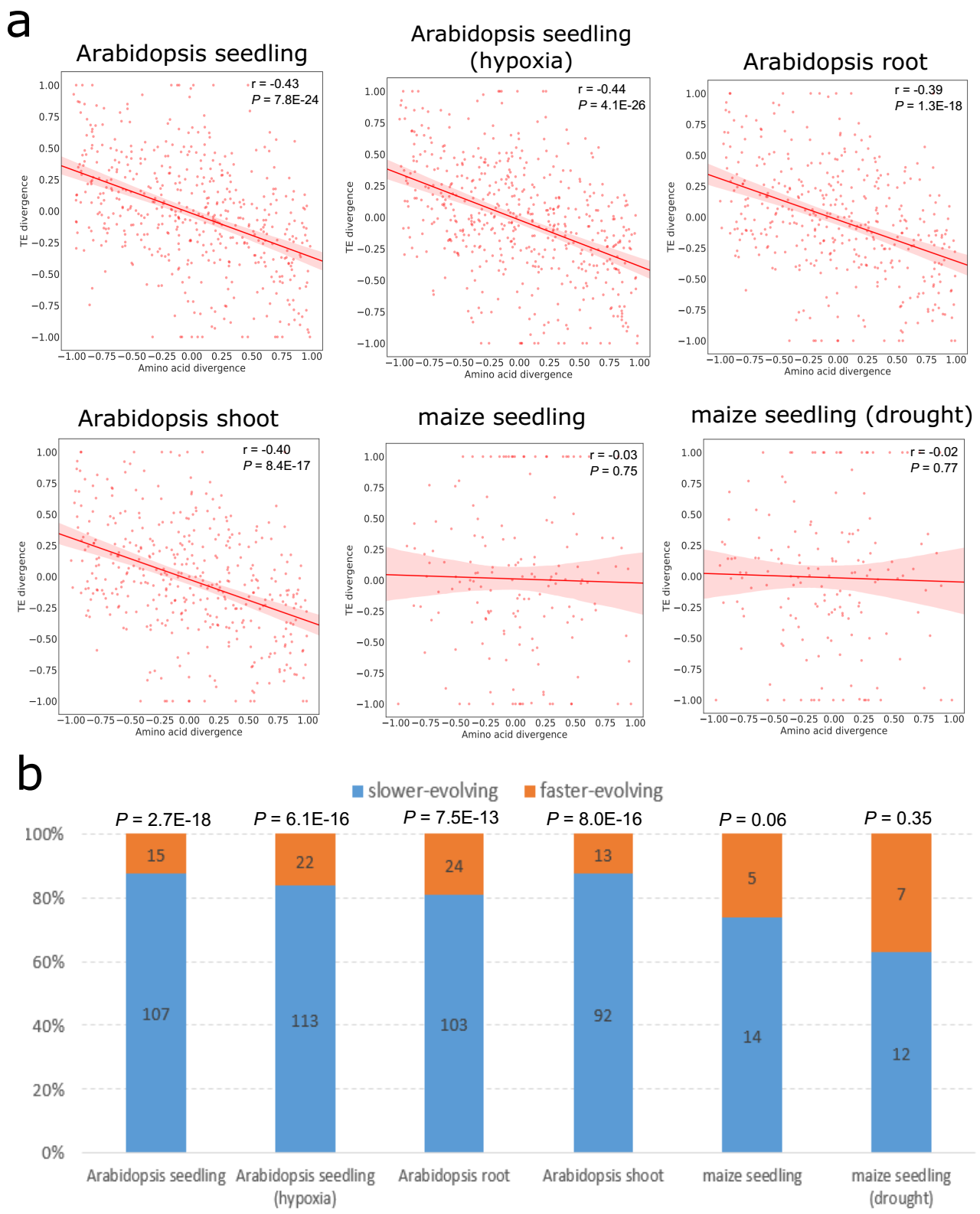


**Fig. S3.** Relationship between RF abundance divergence and amino acid divergence for tandem duplicates. (a) Correlation between the RF abundance divergence and amino acid sequence divergence. The Pearson’s correlation coefficient and *P*-value are indicated. The shaded areas represent 95% confidence interval in both plots. (b) Proportion of the paralog copy with the higher RF abundance between the slower- and faster-evolving copy in a duplicate pair. The y-axis represents the proportion of the paralog with the higher RF abundance. Bars in blue and yellow denote the slower-evolving and faster-evolving paralog copy, respectively. Numbers of duplicate pairs are given on the bar. *P*-values derived from a binomial test are shown above the bar.


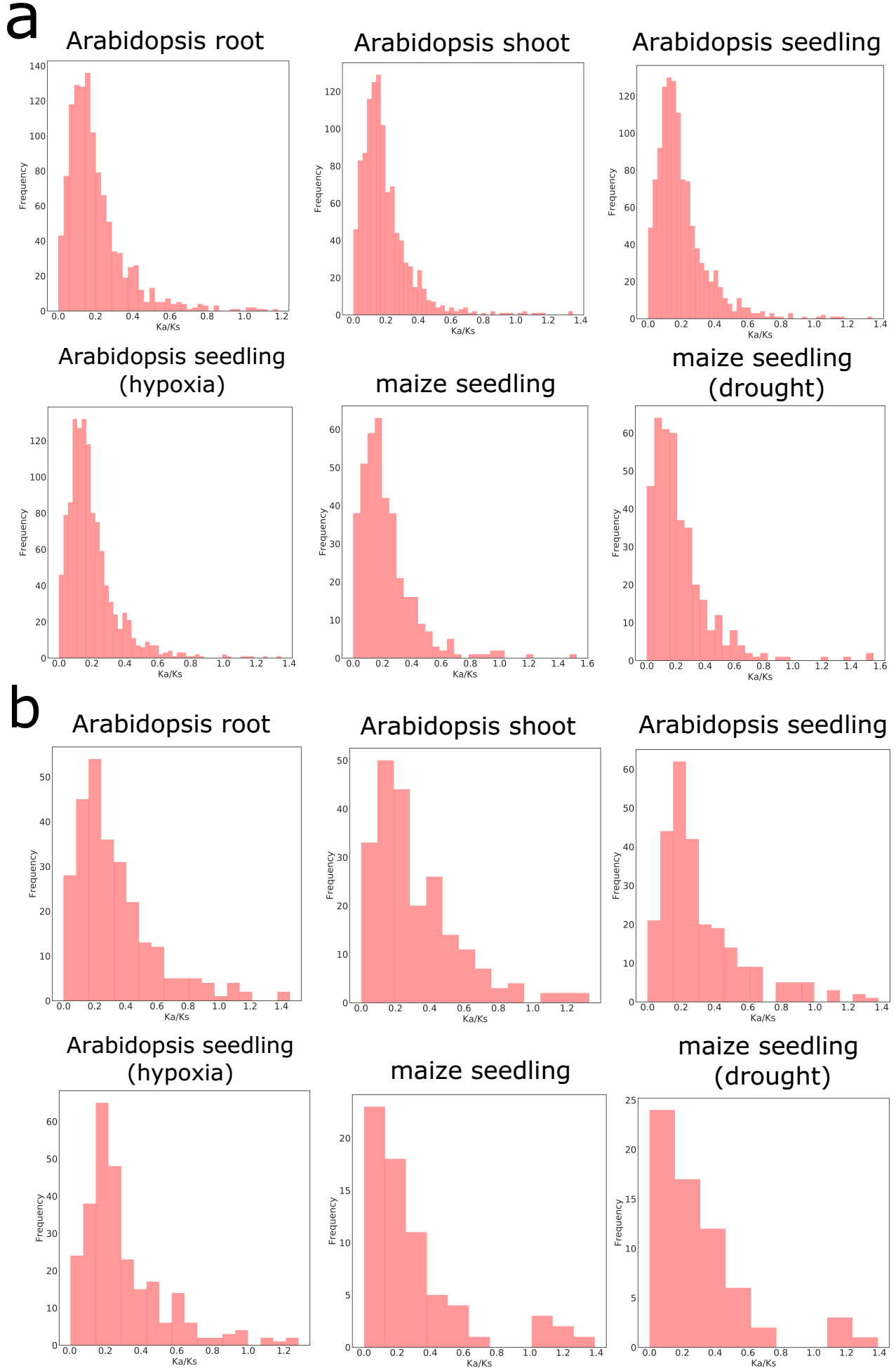


**Fig. S4.** Distribution of the value of Ka/Ks of the paralog copy with the higher RF abundance for WGD (a) and tandem duplicates (b).


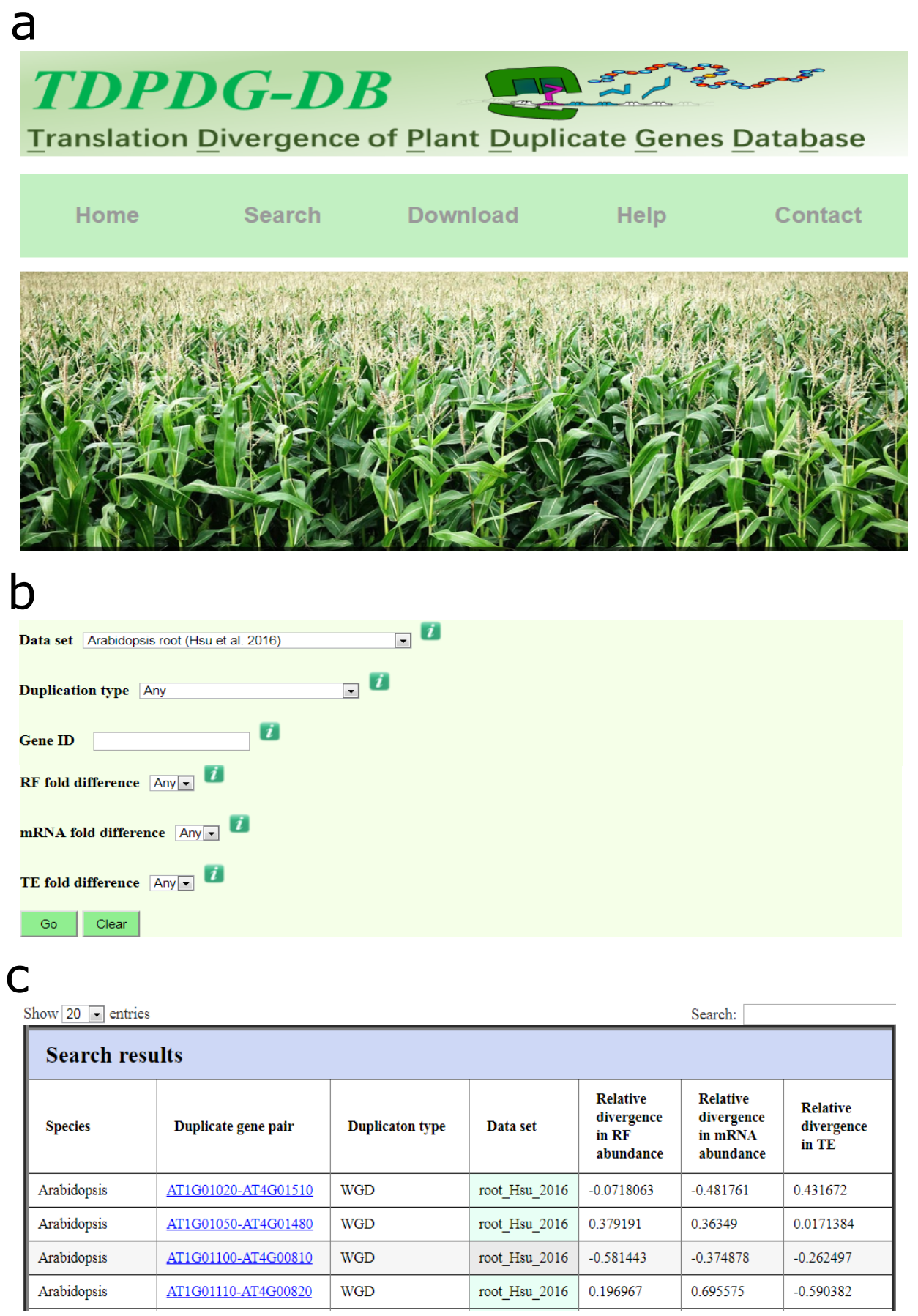


**Fig. S5.** The database for translational divergence of plant duplicate genes. (a) Homepage of the database. (b) The ‘Search’ interface of the database. (c) An examples of the search results.
